# Solar-Supplied Satellite Internet Access Point for the Internet of Things in Remote Areas

**DOI:** 10.3390/s20051409

**Published:** 2020-03-04

**Authors:** Angus Wong, Yan Tai Chow

**Affiliations:** 1 Department of Technology, School of Science and Technology, The Open University of Hong Kong, Hong Kong SAR, China; 2 PCCW Global, Hong Kong SAR, China; jebchow1234@gmail.com

**Keywords:** satellite internet, IoT, solar supply, solar panel, Wi-Fi, geosynchronous equatorial orbit (GEO) satellite networks, low earth orbit (LEO) satellite networks, remote areas

## Abstract

As satellite communications provide ubiquitous coverage, they play a key role in providing Internet connectivity in remote or marginalized areas, so as to enable the vision of a truly global connectivity of the Internet of Things (IoT). However, these areas often lack reliable electricity supply. Thus, this paper proposes a satellite internet access point powered by solar energy, so that a stable Internet connection can be provided. The access point provides Wi-Fi coverage so that sensors, IoT, and devices can connect to the access point using the Wi-Fi, a common wireless technology. Our design took some cost-saving measures to make it affordable and selected the components that require minimal maintenance operations. The satellite access point costs about USD $500, and can provide four days of Internet connectivity without solar energy.

## 1. Introduction

Sensors and the Internet of Things (IoT) can be used to implement various kinds of applications in remote areas, such as collecting environment data and monitoring borders [1]. Many remote areas in developing countries [2] have no access to the internet, which limits the vision of a truly global connectivity of IoT.

Because satellite communications provide ubiquitous coverage, they play an important role in providing internet connectivity in remote or marginalized areas. Studies have shown [3] that using optical fiber or telephone lines to form a network in those areas are problematic. As concluded in another study [4], satellite broadband reaching rural areas is more cost-effective than other technologies. In Bolivia, the government has chosen satellites to fill the gap in universal Internet access. Satellites help some Bolivians in remote areas use the internet. While using fiber optics in Bolivia costs about USD $3000 per 5 km, satellite methods are more affordable and simpler [4].

This article aims to propose a satellite-based internet access point suitable for remote areas with the following objectives:

- To be solar-powered, so it can operate outside the grid. This is particularly important because electricity supply in remote rural areas is often volatile;
- To provide a wireless hotspot so that sensors, IoT, and devices can connect to satellite networks with ease;
- To use low-cost equipment to make the system affordable;
- To be easy to maintain, set up, and use.

Currently, personal satellite terminals are not designed to support multiple sensor or devices. In our design, a satellite phone is connected to a Wi-Fi hotspot module to provide Wi-Fi coverage for the sensors or devices. On the other hand, the cost of satellite communication is not low, and most satellite data plans are charged per megabyte. In this paper, we design an access point with cost in mind and make it affordable.

The work in [5] is similar to our design, i.e., using wireless local network and satellite internet. However, unlike our design, the design in [5] requires an electricity supply. On the other hand, the design in [6] uses solar energy to power satellite terminals, but the work is mainly to demonstrate how the system works and does not cover practical design issues. Unlike previous works, this study provides a practical approach for designing a solar-supplied satellite access point, covering methods to determine the required solar panel size and calculate the number of days to fully charge the battery.

### 1.1. Satellite Phones

There are many options for satellite receivers. You can choose between geosynchronous equatorial orbit (GEO) or low earth orbit (LEO) [7], both of which have advantages and disadvantages. Generally, GEO satellites have the advantage of uninterrupted coverage compared to LEO satellites. However, GEO-based phones use more energy when amplifying signals, and because satellites are placed above the earth [7], they cause more delay. There are four major satellite network companies on the market that provide customers with global services. They are Iridium, Globalstar, Inmarsat, and Thuraya [7]. Thuraya has provided a comparison sheet on six popular satellite phones [8] in terms of form factor, navigation system, reliability, and price. The phones compared are

1. Thuraya XT-PRO DUAL
2. Thuraya XT-PRO
3. Thuraya XT-LITE
4. Iridium 9555
5. Iridium 9575
6. IsatPhone 2

### 1.2. Service Plan

One the other hand, the service plans of the SIM cards for satellite phones have to be considered. Table 1 compares several popular prepaid subscriber identification module (SIM) cards [9]. As can be seen, satellite Internet is inherently low bandwidth (especially when choosing a cheap data plan), so it is recommended to deploy IoT applications with satellite networks for humanitarian relief [10]—for example:

- Issue a disaster warning;
- Request emergency services;
- Push information about the weather;
- Push information related to agriculture and fisheries.

### 1.3. Solar Photovoltaic System

The energy output of a solar photovoltaic (PV) system can be obtained by the following steps [11]:

1. Estimate the area of the photovoltaic panel.
2. Evaluate the tilt angle of the photovoltaic panel.
3. Use data from the U.S. National Aeronautics and Space Administration (NASA) [12] to evaluate solar radiation at latitudes and longitudes.
4. Collect the conversion efficiency of solar photovoltaic panels and calculate the output of the panels in kilowatts.
5. Take the average of the temperature at that location, and then calculate its impact on the photovoltaic panel.
6. Use Equation (1) to calculate the solar panel’s energy output:
  *Solar Panel Output* = *Solar Array Area* × *Conversion Efficiency* × *Solar Radiation* (1)

The costs of different types solar photovoltaic tables were gathered in an online store [13] and are summarized in Table 2.

## 2. System Design

### 2.1. System Components

**Figure 1:**
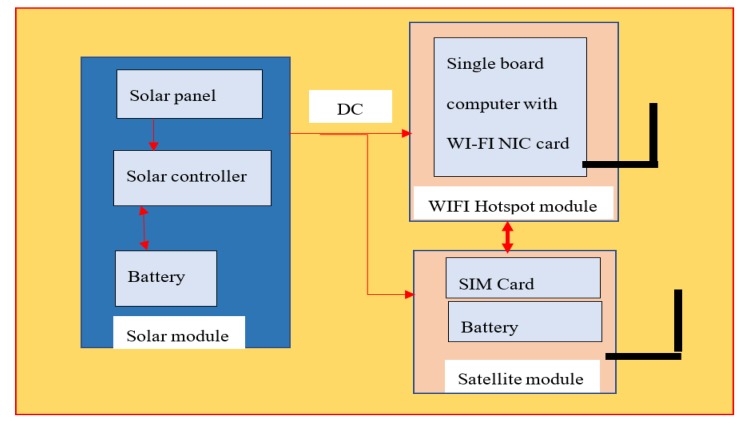
Hardware components of the proposed solar-supplied satellite access point.

The proposed system is comprised of three modules: solar supply, Wi-Fi hotspot, and a satellite. The corresponding system diagram is shown in Figure 1.

- Solar module: this consists of a solar panel that provides energy to the entire access point. The solar controller is used to convert the voltage generated by the solar panel to charge the battery. The battery can be used as a buffer to provide continuous power at night or on cloudy days.
- Wi-Fi hotspot module: this consists of a single-board computer with a Wi-Fi network antenna, which acts as a Wi-Fi hotspot and provides internet connectivity to the local users (via the satellite module).
- Satellite module: this consists of a satellite phone with a SIM card inserted to connect to a satellite network.

### 2.2. Design Considerations for Solar Module

There are two issues to consider in the design of the solar module:

1. The size of solar panel is large enough to meet both Wi-Fi access points and satellite phone power requirements.
2. The system will also be able to maintain power for a certain period of time at night or on cloudy days.

To address the issues, we first calculate the initial estimation of power consumption for the system. Suppose that

- Single-board computer consumption is 5 W (requires a practical investigation on the operating power consumption);
- Satellite phone (Hughes 9201) consumption is 1.36 W;
- Estimated system conversion loss = 40% (loss from the solar controller, cable, voltage converter).

The power output required to sustain the system in maximum load can be obtained by

*Solar Module Output* = *Solar Panel Output* × *Conversion Efficiency* (2)

That is,

(1.36 W + 5 W) / (1% − 40%) = 10.6 W

Thus, the total power consumption by running the access point 24 h is

10.6 W × 24 h = 254.4 Wh

Based on the total power consumption, we can estimate the surface size of the solar panel, which also has excess power that can charge the discharged battery when powering the access point. The solar panel will have following surface size to meet requirements:

The data from NASA website [12] shows that, given a 22 degree tilted tile, the average power per day is 3.15 kWh/m^2^/day. Supposing that typical solar panel efficiency is 18%, the required power per day becomes

3.15 kWh/m^2^/day × 18% = 0.55125 kWh/m^2^/day

Thus, the required solar panel surface is

(254.4 Wh/day) / (551.125 Wh/m^2^/day) = 0.4616 m^2^

In other words, solar panels need a size of at least 0.4616 m^2^ to keep the system fully loaded. The battery used to store the energy of the solar panel should be no less than 254.4 Wh to maintain a day of operation.

### 2.3. Design Considerations for Wi-Fi Hotspot Module

The main design consideration of the Wi-Fi hotspot module is to choose a single-board computer with lower power consumption. The less power an access point consumes, the longer it can connect people to the internet when solar power is not available.

The single-board computer is responsible for hosting the software access point, which broadcasts Wi-Fi signals for users to connect. The access point will act as a network layer router, forwarding user packets to the satellite terminal. The hotspot will run network address translation (NAT) to allow multiple users on the local area network to share the satellite connection. In addition, a dynamic host configuration protocol server (DHCP) will be hosted on the computer to automatically assign Internet protocol (IP) addresses to the local hosts.

### 2.4. Design Considerations for the Satellite Module

The main design choice for satellite phones will be the GEO satellite system, because the system uses satellites fixed in a position in the sky; therefore, there is no need to reconfigure the antenna pointing direction.

Satellite phones were selected instead of a satellite broadband terminal (VSAT) in this paper. Although most VSATs are designed to be stationary and maximize performance, satellite phones are designed to have balanced performance and size when working outdoors. A smaller size and lighter weight can simplify transportation, and the terminal could be more concealed. Although the cost of VSAT’ s high usage plan will be lower than that of satellite phones, the capital cost of VSAT will be higher, ranging from USD $3000 to more than USD $10,000 [14]. Another problem with VSAT is that, unlike satellite phones with global coverage, VSAT equipment can only be used in limited locations.

## 3. Implementation

**Figure 2:**
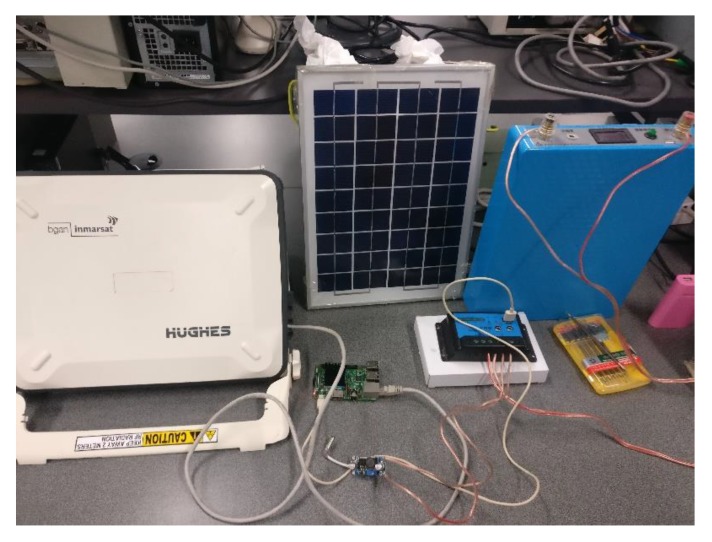
Implementation of the designed solar-powered satellite access point.

This section focuses on the implementation of the proposed solar-powered satellite access points. The prototype is shown in Figure 2.

### 3.1. Solar Module

**Figure 3:**
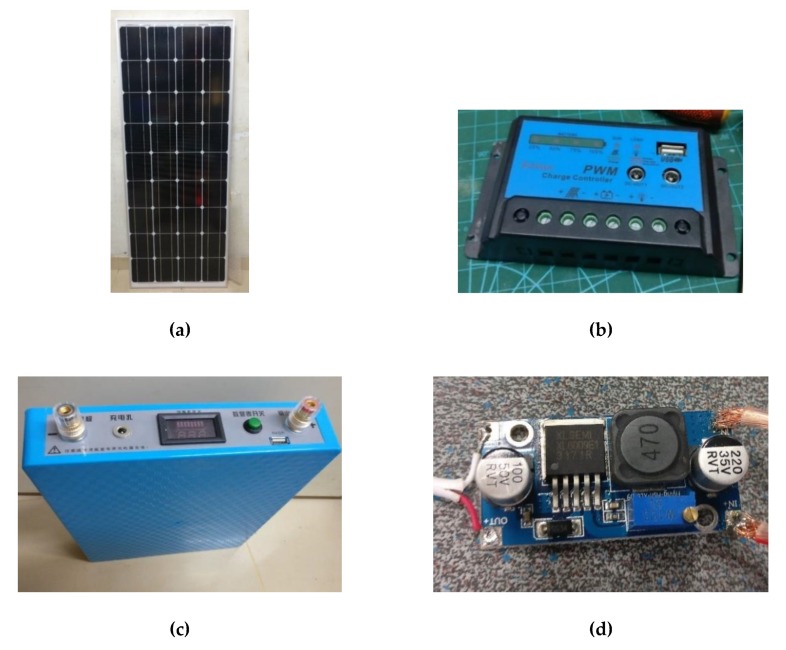
Components in solar module. (**a**) The solar panel that provides the electricity to the system. (**b**) The pulse width modulation (PWM) solar controller that converts the electricity to the system. (**c**) The 720 Wh battery. (**d**) The XL6009E1 DC boost converter.

A 100 W solar panel (shown in Figure 3a) was used to build a solar power source, which was connected to a solar controller (shown in Figure 3b), and powered the entire system, including wireless access points and satellite terminals. The 100 W solar panel was chosen because it is sufficient for providing the energy of the system, and is the most cost-effective in terms of output cost per watt while also powering the satellite access point.

A 720 Wh lithium-ion battery (as shown in Figure 3c) was selected to store the power generated by the panel. The 720 Wh battery has the advantage of being widely used and has a good price/performance ratio (Wh/cost). Battery capacity allows the use of solar energy for at least three consecutive days, which can be beneficial for events such as the rainy season or failure of a solar panel system.

The pulse width modulation (PWM) solar charge controller was selected because it costs less than the cost of a maximum power point tracking (MPPT) charge controller with the maximum acceptable power loss, which can transmit still more power from the solar panel to the access point.

The solar system is expected to be exposed to outdoor weather. The solar system was built to withstand vertical dripping, and the manufacturer of the selected solar panel evaluated the solid particle protection level (level 5) and liquid ingress protection level (level 5) of the International Electrotechnical Commission’s (IEC’s) 60529 [15] standard. The solar panel was connected through a waterproof MC4 connector.

The output voltage of the solar system was equal to the voltage of a 720 Wh lithium-ion battery, with a range of 10.0 V to 12.6 V. The satellite terminal and single-board computers required stable voltage from the solar system. Therefore, a direct current to direct current (DC–DC) voltage converter module was required. The PWM solar charge controller had a USB output that provided 5 V and 2 A power. However, the satellite terminal required 19 V to operate, so the XL6009E1 DC boost converter module (shown in Figure 3d) was connected to the solar system to provide power to the satellite terminal.

### 3.2. Wi-Fi Hotspot Module

A single-board Raspberry pi 3 model B (RP3B) computer was selected. This is because the hardware uses a low-power design quad-core processor that minimizes power consumption but is powerful enough to host access point applications. It is also cheaper than those single-board computers that run an x86 system. The RP3B model is the first Raspberry Pi that has an integrated Wi-Fi network interface card (NIC), allowing the software on the single board computer to run Wi-Fi hotspot service.

The Raspberry Pi was installed with Raspbian stretch Operation System, which is a Raspberry Pi distro. The system was installed the following software packages, which are used to set up the Wi-Fi Hotspot on Raspberry Pi.

- Dnsmasq: provides domain name system (DNS) and DHCP services;
- Hostapd: manages and broadcasts Wi-Fi signal via the wireless network interface card. In this study, the service set identifier (SSID) assigned is “satnet.”

### 3.3. Satellite Terminal

Inmarsat (as shown in Table 1) was selected as the satellite internet service provider in this study, and its Broadband Global Area Network (BGAN) service plan is used. BGAN uses GEO systems that do not require antenna redirection, so there is no need to maintain antenna pointing. Inmarsat’s BGAN service plan covers the whole world without additional charges and reconfiguration, making the system universal in every country. Another reason to choose Inmarsat BGAN over other satellite phones is that it offers the fastest connection speeds and flexible data usage plans [16] with a cost as low as USD $65 for 20 MB.

The Hughes 9201 satellite terminal [17] was chosen because it is the last generation system with a sharp price drop. Compare it to the latest generation system (Hughes 9202 BGAN Satellite Terminal [18]), which costs USD $2795, the 9201 terminal costs only USD $350. The Hughes 9201 offers the same internet speed and similar connection speeds.

### 3.4. Enclosure

**Figure 4:**
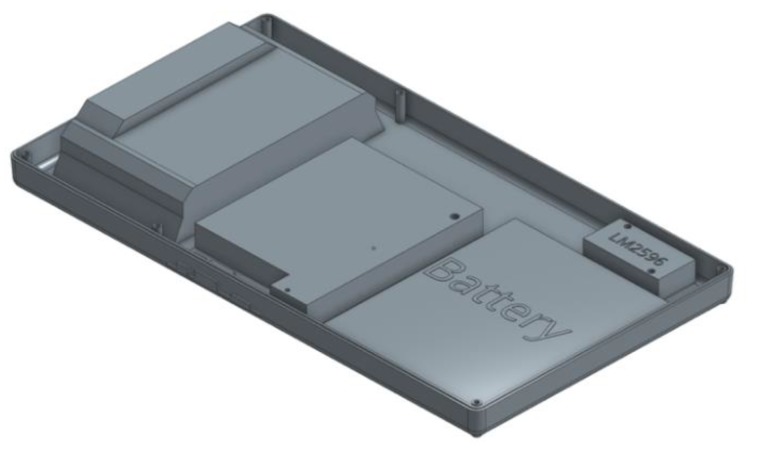
The three-dimensional (3D) rendered enclosure for housing the solar controller, single-board computer, and DC boost converter.

The enclosure encloses the device, which can face various challenges, such as liquid damage from weather. The case size was estimated by using computer-aided design (CAD) software, as shown in Figure 4. This enclosure was used to house the PWM solar controller, the single-board computer, and the DC boost converter. This enclosure could be installed under the solar panel to provide further liquid protection for the device. Waterproof enclosures could be purchased to protect internal electronics. The box was estimated to be 270 mm in length, 150 in width, and 35 mm in height, using acrylonitrile butadiene styrene (ABS) as the material.

### 3.5. Cost of Hardware

The total capital cost (see Table 3) is USD $504, which excludes installation and assembly costs. Solar panels and PWM solar controllers were ordered directly from the factory via an e-commerce platform. Operational costs are dependent on the use cases—whether users more targeted by SMS (see Table 4) or by email (see Table 5). The monthly plan of the Inmarsat BGAN network can accommodate 20 MB of data, while SMS sent by satellite terminals is charged. Each SMS sent by the prepaid SIM card will consume 0.5 units, and the prepaid SIM card will expire every three months.

## 4. Results and Evaluations

The satellite access point based on our design (see Figure 3) was built and tested. We evaluated the power consumption of the system and verified the parameters of the solar module.

### 4.1. The Power Consumption of the System

The power consumption of the single-board computer was measured by running it for one hour. The power consumption of the satellite terminal was determined by a current clamp meter that probed the cable connecting the battery to the PWM charge controller. The single-board computer was connected to the USB of the PWM charge controller through a USB tester, which was connected to the 720 Wh lithium-ion battery of the system to fully simulate the actual situation. The measurement results are shown in Table 6.

### 4.2. The Pulse Width Modulation Charge Controller Efficiency Loss from Charging

The efficiency of the PWM charge controller is critical to determining the total amount of power the solar module will generate. The PWM charge controller cannot reach the maximum power point, because it can operate linearly by controlling the voltage of the solar panel [19]. The efficiency loss can be calculated using the maximum power point provided by the manufacturer, which indicates that the voltage at the maximum power point is 18 V, and the nominal voltage of the three-cell lithium-ion battery is 11.1 V.

Therefore, the nominal efficiency loss of the PWM charge controller was estimated by

(The voltage at maximum power point − nominal voltage/nominal voltage) × 100% = 62%

### 4.3. Results of the Solar Module

The solar modules were evaluated using five parameters. The results are shown in Table 7. These parameters were evaluated according to the day of the month with the largest average energy output and the one with the smallest average energy output.

Suppose that the solar panel was located at a latitude of 22° and a longitude of 114°, and was tilted 22 degrees, as determined by data from the surface meteorology and solar energy database from NASA [12]:

- the largest radiation of month = 5.6 (kWh/m^2^/day);
- the smallest radiation of month = 4.63 (kWh/m^2^/day).

Therefore, the results were calculated based on the above maximum and minimum radiations and are shown in Table 7:

1. **Solar panel output:** as obtained from the manufacturer’s data sheet: panel conversion efficiency = 18%; solar array area = 0.5625 m^2^. Thus, the solar panel output can be obtained by Equation (1).
2. **Solar module output:** As shown in Equation (2), to calculate the solar module output, the conversion efficiency has to be known, which is the efficiency of PWM solar energy to convert solar panel energy into electrical energy. This is estimated based on the efficiency loss of the charging process of the PWM charging controller. As calculated in Section 5.2, the conversation efficiency is 62%. Thus, the solar module output can be obtained by Equation (2).
3. **System power consumption:** this is the sum of the power consumption of the single-broad computer and the satellite terminal for a day. By measurement, as shown in Table 6, the power consumption can be obtained by (5.2 + 1.08) W × 24 h = 150.72 Wh/day.
4. **Energy surplus:** the energy surplus per day can be determined by (solar system output – system power consumption).
5. **Time to fully charge battery:** the time it takes for the battery to fully charge can be determined by battery capacity/energy surplus per day. In this study, the factory-rated battery capacity was 720 Wh.

### 4.4. Evaluation of the Solar Module

As can be seem from Table 7, the solar module can generate enough power from the solar panel to meet the needs of the access point (powering the Wi-Fi hotspot module and satellite module). On the other hand, during the month with the largest radiation, it takes only 5.41 days to fully charge the battery while the system is in production. As the battery capacity is 720 Wh, and the system power consumption is 150.72 Wh/day, the system can last 720/150.72 = 4.78 days, even without solar energy. As a result, the proposed solar-powered satellite access point can be self-sustaining without an external power source, and is suitable for deployment in remote areas where electricity supply is unstable.

There were limitations to the evaluation of solar panels and PWM solar controllers. Solar panel evaluation relied on the data sheet provided by the manufacturer, which might not match the actual characteristics of the solar panel. The charging efficiency of the PWM solar controller was estimated based on the characteristics of the PWM solar controller, and not by measurement. To determine the charging efficiency of a solar controller, a solar array simulator is needed, which is beyond the scope of this paper.

### 4.5. Validation of Wi-Fi Hotspot and Internet Connection

To verify the internet connection provided by the satellite access point, we used a laptop (running Windows 10) to connect to the Wi-Fi hotspot (provided by the satellite access point). We observed that once the laptop was started, it was connected to the Wi-Fi network with the SSID “satnet” (as described in Section 4.2), and a private IP address was assigned by the DHCP server in the single-broad computer. After that, we successfully accessed a web page through the Internet.

## 5. Discussions

### 5.1. LEO and Its Potential in Future Internet of Things Services

In this paper, INMARSAT GEO was used. Note that using GEO-based service is only one possible approach to implement the proposed satellite access point. There are LEO satellite constellations that are currently being deployed or designed to provide global IoT connectivity, which are likely to achieve lower costs and higher throughput than that of our system. For example, SpaceX, a private American aerospace manufacturer and space transportation services company, has recently submitted filings to the International Telecommunication Union to arrange a spectrum for 30,000 additional Starlink satellites (LEO-based) [20]. SpaceX expects its constellation to have 23−35 ms of latency and up to a gigabyte of throughput. As predicted by the CEO of Telesat, a Canadian satellite communications company, the deployment of these new satellite systems will significantly reduce the price of IoT services via satellite over the next few years [21].

### 5.2. Costs of Implementation

While the costs of equipment and data plans are detailed in Table 3, Table 4 and Table 5, other costs must also be considered in actual deployment. The costs include

1. Arrangement for initial installation: since the proposed satellite access point is provided by solar energy and uses a satellite phone as a terminal, its installation is simple. As long as it is placed in a location of interest and powered on, the Wi-Fi hotspot and satellite connection will be ready accordingly. Therefore, the cost of the initial installation is one-time and minimal.
2. Need for remote maintenance services: as there is a single-board computer in the proposed satellite access point, remote configuration for the hotspot settings is feasible. However, if unexpected hardware errors occur, arrangement for the repair or maintenance work is required.
3. Arrangement for the renewal of SIM cards: if prepaid SIM cards are used, arrangements are required for their renewal. To reduce this kind of arrangement, 365-day prepaid SIM cards or monthly (or even yearly) service plans and automatic payments can be considered.
4. Fee to national agencies: in some countries, a fee has to be paid to the national agencies responsible for satellite services.

Even though the above costs are required, they are lower than other methods (e.g., VSATs mentioned in Section 2.4 and cabling a physical network mentioned in Section 1), and the proposed satellite access point is a feasible approach in providing internet connectivity to the IoT in remote areas.

### 5.3. Modular Design to Cater to other Communication Modules

The proposed satellite access point consists of three modules: solar, satellite, and the Wi-Fi hotspot, as shown in Figure 1. Instead of Wi-Fi, to provide communication to IoT, the proposed system can be extended to support other communication technologies, such as LoRaWAN [22] or Sigfox [23]. In the solar module, a solar controller is used to convert the voltage generated by the solar panel to DC output. The DC output can be used to power other communication modules. For example, if a LoRaWAN module is used, the solar-supplied satellite terminal become the Internet gateway for the LoRaWAN networks. The LoRaWAN clients (e.g., IoT equipped with a LoRaWAN interface) can then connect to the LoRaWAN module and access the remote IoT server (through the solar-supplied satellite terminal).

## 6. Conclusions

A satellite access point powered by solar panels was developed to provide communication services to the sensors or IoT in remote areas. The advantage of this design is that it can be easily set up, maintained, and deployed anywhere. Because it is solar-powered, it can be fully self-sustaining without user intervention. Moreover, it is low-cost. For about USD $500 in equipment costs, an ad hoc infrastructure can be established in remote or marginalized areas.

## Figures and Tables

**Table 1:** The price of a prepaid SIM card for connecting to a satellite network.

Card	Entry Plan Cost (USD)	Cost (USD) per MB/mins	Connection Speed	Free Short Message Service (SMS)
IsatPhone Pro (GEO)	$50 (50 unit)	$1/min	2.4 kbps	Yes
Iridium GO (LEO)	$600 (400 unit)	$1.5/min	2.4 kbps	Yes
Iridium Phone	$100 (50 unit)	$2/min	2.4 kbps	Yes
Thuraya Pre-Paid	$70.00 (20 unit)	$17.5/MB	60 kbps (GmPRS)	N/A
Inmarsat BGAN	$50 (50 unit)	$3.6/min	32 kbps	Yes

**Table 2:** A table of the prices of solar photovoltaic panels gathered from the internet.

Type	Monocrystalline (>18%) (USD)	Polycrystalline (>15%) (USD)	Cost per Watt (USD)
10 W	$11.74	$5.27	$0.594
20 W	$11.29	$11.89	$0.376
30 W	$16.26	$11.29	$0.391
40 W	$21.83	$15.65	$0.406
50 W	$21.06	$20.322	$0.361
100 W	$39.49	$36.129	$0.594

**Table 3:** The capital cost of the access point.

Items	Price (USD)
Hughes 9201 INMARSAT BGAN Terminal	$361
100 W solar panels	$37
FGWX 12 V 60 AH li-ion battery	$62
Raspberry Pi 3 model B	$35
PWM solar controller with USB	$4
Enclosure	$5
Total	$504

**Table 4:** The operation cost per 3 months of using a prepaid SIM card.

Item	Price (USD)
Satcom Inmarsat BGAN 50-unit Prepaid SIM card	$115

**Table 5:** The operation cost of using a monthly plan for under 20 MB of data usage.

Item	Price (USD)
Outfitter Satellite Inmarsat BGAN Standard PLUS	$65 ^1^

^1^https://fs30.formsite.com/OutfitterSat/form31/index.html.

**Table 6:** The power consumption of the devices.

Devices	Power Consumption in an Hour
Satellite terminal	5.2 W
Raspberry Pi	1.08 W

**Table 7:** The parameters for the solar module.

Parameter	Largest Radiation	Smallest Radiation
Solar Panel Output	486.94 Wh/day	332.34 Wh/day
Solar Module Output	288.16 Wh/day	196.67 Wh/day
System Power Consumption	150.72 Wh/day	150.72 Wh/day
Energy Surplus	132.85 Wh/day	41.36 Wh/day
Time to Fully Charge Battery	5.41 days	17.40 days
